# Survival of patients with operable breast cancer (Stages I-III) at a Brazilian public hospital - a closer look into cause-specific mortality

**DOI:** 10.1186/1471-2407-13-434

**Published:** 2013-09-24

**Authors:** Débora Balabram, Cassio M Turra, Helenice Gobbi

**Affiliations:** 1 Breast Pathology Laboratory, School of Medicine, Federal University of Minas Gerais (UFMG), Belo Horizonte, Brazil; 2 Department of Demography, Center for Development and Regional Planning, (Cedeplar), Federal University of Minas Gerais (UFMG), Belo Horizonte, Brazil

**Keywords:** Breast neoplasms, Survival analysis, Neoplasm staging, Brazil, Cohort study

## Abstract

**Background:**

Breast cancer incidence is increasing. The survival rate varies and is longer in high-income countries. In Brazil, lower-income populations rely on the Unified Public Health System (Sistema Único de Saude, SUS) for breast cancer care. The goal of our study is to evaluate the survival of patients with operable breast cancer stages I-III at a Brazilian public hospital that treats mostly patients from the SUS.

**Methods:**

A cohort study of patients who underwent surgery for breast cancer treatment at the Clinical Hospital of the Federal University of Minas Gerais from 2001 to 2008 was performed, with a population of 897 cases. Information on tumor pathology and staging, as well as patients’ age and type of health coverage (SUS or private system) was collected. A probabilistic record linkage was performed with the database of the Mortality Information System to identify patients who died by December 31th, 2011. The basic cause of death was retrieved, and breast cancer-specific survival rates were estimated with the Kaplan-Meier method. The Cox proportional hazards model was used for univariate and multivariate analysis of factors related to survival.

**Results:**

A total of 282 deaths occurred during the study’s period, 228 of them due to breast cancer. Five-year breast cancer-specific survival rates were 95.5% for stage I, 85.1% for stage II and 62.1% for stage III disease. Patients from the SUS had higher stages at diagnosis (42% was in stage III, and from the private system only 17.6% was in this stage), and in the univariate but not multivariate analysis, being treated by the SUS was associated with shorter survival (hazard ratio, HR = 2.22, 95% CI 1.24-3.98). In the multivariate analysis, larger tumor size, higher histologic grade, higher number of positive nodes and age older than 70 years were associated with a shorter breast cancer-specific survival.

**Conclusions:**

Five-year breast cancer survival was comparable to other Brazilian cohorts. Patients treated by the SUS, rather than by the private system, had shorter survival times, mostly due to higher initial stage of the disease.

## Background

Breast cancer is the most common malignant neoplasm among women in the world. The incidence is increasing, especially in low and middle-income countries [1]. In 2012, the incidence of breast cancer was expected to be 52.5 per 100,000 women in Brazil [2], whereas the age-adjusted mortality was 11.5 deaths per 100,000 women in 2009 [3]. In high-income regions, population-based studies show higher survival rates [4]: for patients diagnosed between 1990 and 1994, 5-year relative survival was 83.9% in the United States (US) and 73.1% in Europe [4]. In low-income countries, shorter overall survival has been documented, being as low as 38.8% in Sétif, Algeria, for patients diagnosed in the same period [4]. In Goiania, located in the central-west region of Brazil, the survival rate was 65.4% [4].

A patient’s survival is related to several prognostic factors, including number of positive lymph nodes, tumor size, hormone receptor status, histological type and grade, and patient’s age [5]. Socioeconomic status is known to be an intervening factor, mostly because of lower frequencies of patients undergoing interval screening, treatment’s delay and smaller availability of modalities of treatment, such as chemo, hormone, and radiotherapy, among the less affluent populations [6-9].

In Brazil, most of the population does not have private health insurance, and relies on the Unified Public Health System (Sistema Único de Saúde, SUS) for care, which provides patients with screening, diagnosis, and breast cancer treatment [10,11]. In 2008, only 26% of the Brazilian population had private health insurance [11].

Studies from Brazil and other countries were retrieved from the PubMed and LILACS databases in February 14, 2013, using the search terms breast cancer, survival, and Brazil. Seven hospital cohort studies that separated patients by stage and were not aiming to evaluate specific prognostic markers or new treatments were selected. For PubMed, English language was used, and for LILACS, both the English and Portuguese languages were used. Findings from these observational cohorts in different Brazilian hospitals suggested that 5-year breast cancer-specific survival rates have ranged from 90% to 97% for stage I, 87.8% to 89% for stage II and 51% to 73% for stage III breast cancer diagnosed since the 1990s [6,12-16]. In these studies, the methods used to classify a death as due to breast cancer or its treatment vary, and they are sometimes poorly reported or derived only from the basic cause of death, as reported in patients’ death certificates.

In this article, we present new estimates of survival for Brazilian female patients with operable breast carcinoma (stages I-III). We provide estimates for both overall survival rates and breast cancer-specific survival rates, calculated as the probability of surviving breast cancer in the absence of other causes of death [17]. We also look at the association between several prognostic markers and survival rates. Our data come from patients treated from 2001 to 2008 at the Clinical Hospital of the Federal University of Minas Gerais (Hospital das Clínicas, Universidade Federal de Minas Gerais, HC-UFMG), Belo Horizonte, Brazil. The HC-UFMG is a general teaching hospital that treats mostly patients from the SUS coming from Belo Horizonte (the state’s capital) or from smaller cities without a tertiary health care center [18]. It provides patients with surgery as well as chemo- and endocrine therapies. Radiotherapy is performed at other cancer centers in the city. The Breast Pathology Laboratory of the UFMG School of Medicine is responsible for all breast pathology exams from the HC-UFMG and it has kept records of diagnostic and surgical specimens from it since 1989 [18].

## Methods

### Study’s design

We designed a cohort study of patients with invasive operable breast carcinoma in stages I-III surgically treated at HC-UFMG from 2001 to 2008. The study protocol was approved by the UFMG Ethics Committee on March 7, 2012 (project CAAE number 0660.0.203.000-11).

### Study’s population

The cases were retrieved from files of the Breast Pathology Laboratory of the UFMG School of Medicine. We selected all specimens related to surgical treatment of breast cancer.

Among the 1119 patients who underwent surgery for breast cancer treatment at HC-UFMG from 2001 to 2008, we excluded 166 cases of ductal and lobular carcinoma *in situ*, as well as 2 patients with axillary metastasis only (unknown primary site), 1 patient with unknown tumor stage, 27 patients with unavailable primary tumor sample at our institution (first surgery at another institution, no remaining tumor in re-excision for clear margins), 7 patients with metastatic breast cancer who underwent palliative surgery only, 14 patients who underwent surgery for recurrent breast cancer, 1 patient who moved to a different state while on treatment and 4 patients with missing date of birth and mother’s name. Eight hundred ninety-seven cases were available for the final analysis.

### Variables

In addition to date of birth and type of health plan (private insurance or SUS), we recorded twelve variables related to breast cancer diagnosis and treatment: patient’s age, tumor size (T), regional lymph node status (N), age, laterality (right or left), having bilateral cancer, histopathological type (invasive ductal carcinoma not otherwise specified; invasive lobular carcinoma; and special-type carcinomas), histologic tumor grade (according to the Nottingham grading system) [5], type of surgery performed (mastectomy or breast-conserving surgery), undergoing axillary node dissection, use of neoadjuvant chemo- or hormone therapy, and type of health plan (SUS or private system) [11]. Tumor staging was performed in accordance with the 7th edition of the American Joint Committee on Cancer (AJCC) Cancer Staging Manual [5]. For patients who did not undergo neoadjuvant systemic therapies, pathologic tumor stage (which is the gold standard for cancer staging) was used [5]; in the other cases, clinical tumor stage prior to therapy was used as a surrogate.

### Information on survival status and death causes

We retrieved information on survival status, and date and cause of death from the Mortality Information System (MIS) of the Ministry of Health in Brazil for the years 2001 through 2011. The MIS is a national, computerized index of death record information that was implemented in 1975. Over the years, the completeness of death registration in the MIS has improved substantially, reaching 93.5% as of 2007 in Minas Gerais [19]. Because patients from the HC-UFMG were all residents of the state of Minas Gerais, we restricted the MIS database to the cases who were residing in Minas Gerais at the date of their death. To identify patients from the study cohort who died from January 1, 2001 to December 31, 2011, we linked the MIS death records to the HC-UFMG data. A probabilistic record linkage was conducted using the software RecLink, version 3.0 (http://www.iesc.ufrj.br/reclink/) [20]. The probabilistic method is used when a unique identifier, such as social security number, is unavailable. To reduce the number of possible pairs, after standardizing both databases, we applied a four-step blocking strategy: first, using the soundex code of patients’ first and last names and years of birth; second, using the soundex code of the mothers’ first and last names and years of birth; third, based on soundex code of patients’ and mothers’ first name and years of birth; and fourth with only patients’ first names and years of birth. We then paired the cases within each block, and estimated a linkage score for each pair based on the name and date of birth. All pairs with scores higher than 1 were reviewed in order to confirm them as true or false by using the fathers’ names and addresses. Patients who were not found in the MIS database were presumed to be alive as of December 31, 2011 and therefore censored at this date.

In the Mortality Information System, causes of death are classified according to the International Classification of Diseases, version 10 (ICD 10) [21], by a technician [22].

After reading all causes of death described in each death certificate, we applied the coding by the Surveillance, Epidemiology, and End Results (SEER), of the U.S. National Cancer Institute to estimate breast cancer-specific survival. Cases with unknown death causes were not excluded [17]. When the cause of death was unknown or the patient died without assistance (8 cases, 2.8%), breast cancer was considered to be the cause [23]. When breast cancer was considered to have contributed to death, the patient was classified as having died from the disease (12 cases, 4.3%) [9].

An alternative analysis was performed, considering only the basic cause of death, as selected by technicians from the State’s Secretaries in Health, which is used for national mortality statistics. The methods reported by SEER were also used in this situation.

### Statistical analysis

We estimated Kaplan-Meier curves to describe the survival of this cohort over 5- and 10-year periods. We used the log-rank test to compare the survival distributions of different subgroups in our data. Since the date of the first biopsy was not available for all patients who had surgery as the primary treatment, survival interval was calculated in months from date of surgery in patients who did not undergo neoadjuvant chemo- or hormone therapy and from biopsy date in patients who underwent such therapies. Also, we tried to keep the staging as accurate as possible by using the clinical stage at the date of biopsy or the pathological stage at the date of surgery.

Age was categorized in three subgroups: up to 35 years, 36–69 years, and 70 years and older.

Mean age and standard deviation (SD) were calculated. The chi-square test was used to compare categorical variables. The chi-square test for a linear trend was used to compare the frequencies of tumor stage over the years of the study, as well as tumor stage in each age category. The significance level was defined as 0.05. The Cox proportional hazards model was used for hazard ratio (HR) and 95% confidence interval (CI) estimation in the univariate analysis and for multivariate survival analysis with a stepwise backward conditional strategy. Variables with statistical significance (p < 0.05) in the univariate analysis were initially used for the multivariate model, except for type of surgery, performing axillary node dissection, and use of neoadjuvant therapy, since we had incomplete data on treatment, to avoid biasing the results. For instance, patients diagnosed at higher stages probably underwent adjuvant systemic therapies later on. However, we did not have the data to confirm this information. Only variables with a p value bellow 0.05 were kept in the final multivariate model. All statistical analyses were performed with the SPSS software, version 17.0 (SPSS Inc, Chicago, IL).

## Results

Five-year breast cancer-specific survival for the entire cohort was 78.5%, and 10-year survival was 64.5%. The cause-specific survival was 95.5% at 5 years for stage I, 85.1% for stage II, and 62.1% for stage III disease. Overall survival was 92.1% for stage I, 81.8% for stage II, and 58% for stage III disease. Only a small proportion of our patients were followed over a 10-year period (45 patients, 5%); among those in stage I, 10-year survival rate was 91.2%, 69.8% for stage II, and 43% for stage III patients.

The median period of follow-up was 64 months (range 1–131 months). Among the 897 patients, 282 (31.44%) died during follow-up, out of whom 228 (80.9%) died from breast cancer and 54 (19.1%) from other causes. Cardiovascular diseases (ICD 10 chapter IX) was a frequent cause of death unrelated to breast cancer, with 16 cases (29.6% of other death causes, data not shown). Four patients had unattended deaths (1.42% of total of deaths), and 3 patients (1.06% of total of deaths) had deaths from unknown causes.

Table 1 shows the distribution of patient characteristics, life status at the end of the study period, and HR for the different factors examined in the univariate analyses. The mean age of patients was 55.32 years (SD = 13.97, range 20–97 years), and the median age was 53 years. Only 47 patients (5.24%) were 35 years old or younger; 677 patients (75.47%) were between 36 and 69 years, and 173 patients (19.29%) were 70 and older. Most individuals (823, 91.75%) were treated in the SUS; only 74 (8.25%) were treated in the private health system. Of those, 65 had private insurance and 9 paid for their treatment. Three hundred forty-eight patients had T2 tumors (2 to 5 cm, 38.8%). As for the axilla, 387 patients (43.14%) had negative lymph nodes, while 510 patients (56.86%) had at least one positive node. A great number of patients were in stage III at diagnosis (359 cases, 40.02%). Twenty-nine patients had bilateral breast cancer either concomitantly or at follow-up, that was treated at our institution (3.23%). Left breast tumors were more common (472 patients, 52.6%). Regarding pathologic type, most patients had invasive ductal carcinoma not otherwise specified (760, 84.73%). Seventy-nine patients had invasive lobular carcinoma (8.81%), and 58 patients (6.47%) had other pathologic subtypes. One hundred eighty-one patients had low-grade tumors (20.18%), 385 had intermediate-grade tumors (42.92%) and 320 had high-grade tumors (35.67% of patients). The most common surgery was mastectomy, performed in 59.87% of patients (537 cases). Axillary node dissection was performed in 684 (78.25%) patients. One hundred sixty-six patients (18.51%) underwent neoadjuvant therapies (3 had combined neoadjuvant chemo- and hormone therapy, 4 had hormone therapy exclusively and the other patients had neoadjuvant chemotherapy only).

**Table 1:** Patients’ characteristics and univariate analysis of factors related to survival

**Factor**	**Cases**	**%**	**Events**	**%**	p value*	**HR**	95% CI**
**Age**					.012		
**Up to 35 years old**	47	5.24	16	34.04		1.63	0.98–2.73
**36-69 years old**	677	75.47	159	23.49		1.00	
**70 and older**	173	19.29	53	30.64		1.50	1.10–2.04
**Tumor size**					< 0.001		
**T1 (up to 2 cm)**	319	35.56	33	10.34		1.00	
**T2 (2–5 cm)**	348	38.80	88	25.29		2.59	1.73–3.86
**T3**	105	11.71	37	35.24		4.03	2.52–6.45
**T4**	125	13.94	70	56.00		8.02	5.29–12.16
**Lymph node status**					< 0.001		
**N0**	387	43.14	45	11.63		1.00	
**N1**	255	28.43	67	26.27		2.56	1.76–3.74
**N2**	155	17.28	68	43.87		4.83	3.31–7.04
**N3**	100	11.15	48	48.00		5.25	3.50–7.90
**Stage**					< 0.001		
**I**	223	24.86	13	5.83		1.00	
**II**	315	35.12	58	18.41		3.34	1.83–6.10
**III**	359	40.02	157	43.73		9.84	5.58–17.33
**Bilateral breast cancer**					0.380		
**Yes**	29	3.23	10	34.48		1.33	0.70–2.50
**No**	868	96.77	218	25.12		1.00	
**Histologic grade**					< 0.001		
**Grade 1**	181	20.18	23	12.71		1.00	
**Grade 2**	385	42.92	77	20.00		1.71	1.07–2.72
**Grade 3**	320	35.67	124	38.75		3.72	2.38–5.80
**Unknown**	11	1.23	4	36.36			
**Pathology**		0.00			.449		
**Invasive ductal carcinoma**	760	84.73	199	26.18		1.00	
**Invasive lobular carcinoma**	79	8.81	15	18.99		0.73	0.43–1.23
**Other**	58	6.47	14	24.14		0.86	0.50–1.48
**Public health system**					0.006		
**Yes**	823	91.75	216	26.25		2.22	1.24–3.98
**No**	74	8.25	12	16.22		1.00	
**Neoadjuvant therapy**					<0.001		
**Yes**	166	18.51	77	46.39		2.87	2.18–3.78
**No**	731	81.49	151	20.66		1.00	
**Axillary node dissection**					<0.001		
**Yes**	684	76.25	211	30.85		3.82	2.33–6.27
**No**	213	23.75	17	7.98		1.00	
**Type of surgery**					<0.001		
**Mastectomy**	537	59.87	182	33.89		2.89	2.09–4.00
**Breast-conserving surgery**	360	40.13	46	12.78		1.00	

*Log-rank test.

**95% Confidence interval.

The stage at diagnosis was higher among patients from the SUS (23.1% was stage I, 34.9 stage II and 42% stage III in the public health system, while 44.6% was stage I, 37.8 was stage II, and 17.6% was stage III in the private health system, p < 0.001). The frequencies of stages did not change over the years (p = 0.11, data not shown).

**Figure 1:**
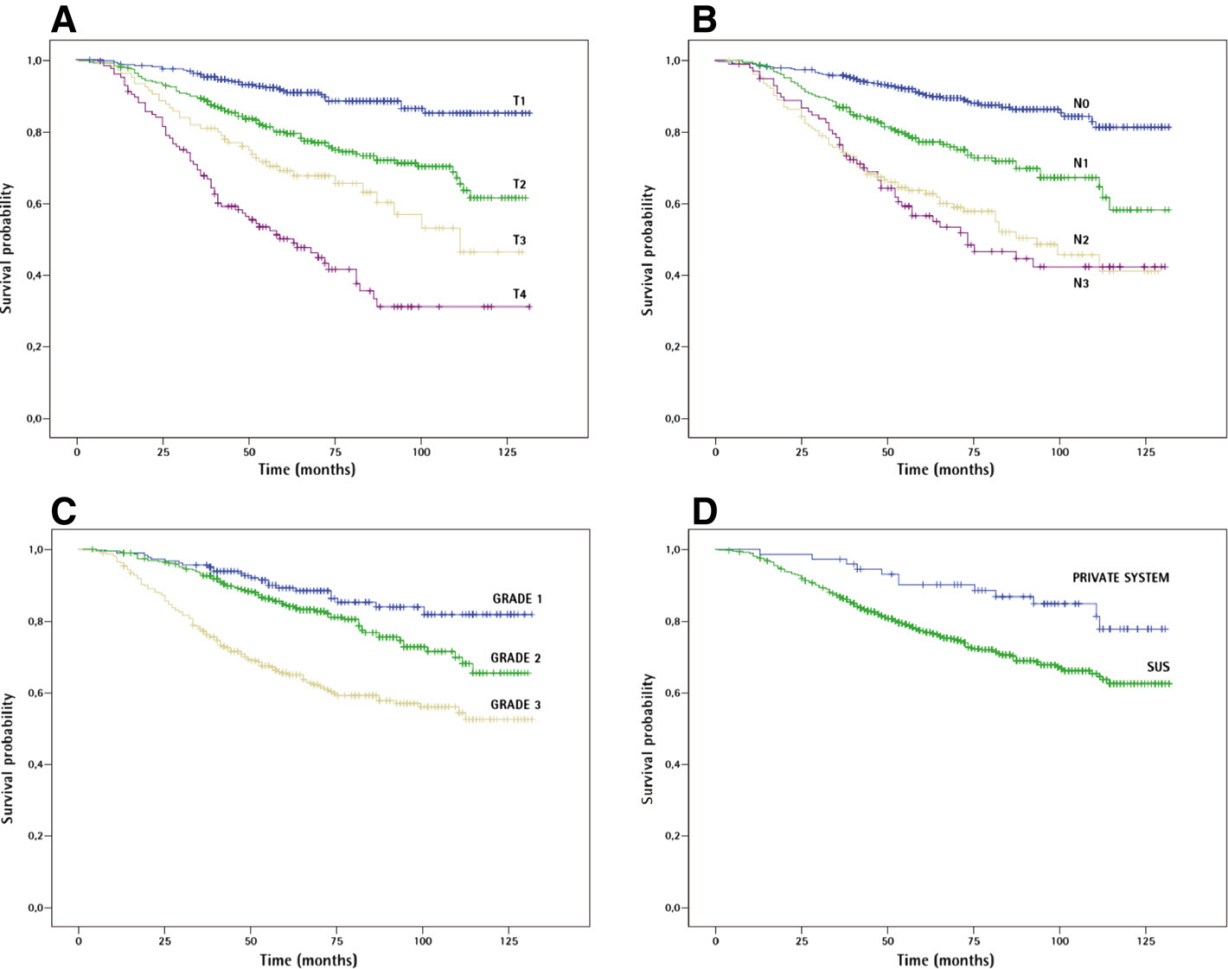
**Kaplan-Meier curves of factors associated with breast cancer survival. A**, breast cancer-specific survival in relation to tumor size (p < 0.001). **B**, survival in relation to lymph node status (p < 0.001). **C**, survival in relation to histologic grade (p < 0.001). **D**, survival in relation to health care system (p = 0.006).

In the univariate analysis, breast cancer in patients older than 70 years of age was associated with significantly lower chances of survival compared to patients 35 to 69 years old. Also, higher histologic tumor grade, larger tumor size, and higher number of involved lymph nodes were associated with lower survival (Figure 1, Table 1). Being treated by the SUS was associated with a shorter survival, with an HR of 2.22 (p = 0.005, CI 1.24-3.98). Older age was not associated with a different stage of disease (p value of Χ^2^ for a linear trend = 0.22) but was associated with a smaller proportion of patients undergoing neoadjuvant systemic therapies (only 8.7% of patients older than 70 years underwent such therapies, whereas 20.8% of patients 36–69 years and 21.3% of patients up to 35 years of age underwent such treatments, p = 0.001, data not shown).

Having bilateral breast cancer and having lobular or special-type carcinomas was not associated with a shorter survival time. In terms of therapy, undergoing neoadjuvant systemic therapy, undergoing mastectomy and undergoing axillary node dissection were associated with shorter survival time, but these variables are highly correlated to tumor stage (Table 1).

In the multivariate analysis, tumor size remained an important prognostic factor. Patients with tumors larger than 5 cm (T3) had an HR of dying due to breast cancer of 2.31 (CI 1.41-3.80) compared to patients with tumors measuring up to 2 cm (T1, Table 2). In addition, patients with tumors infiltrating the skin or chest wall had an HR of 4.34 (CI 2.77-6.79) in relation to T1 patients. Patients with 9 or more positive axillary lymph nodes had an HR of 3.59 (CI 2.35-5.48) in relation to patients with negative nodes. Also, patients aged 70 years and older had a shorter survival (HR in relation to women 36–69 years old, 1.64; CI 1.19-2.26). Patients with high-grade tumors had an HR of 2.54 (CI 1.62-3.96) in relation to patients with low-grade tumors. Being treated by the SUS was not associated with a shorter survival in multivariate analysis.

**Table 2:** Multivariate survival analysis – final model

**Factor**	**p value**	**HR**	**95% CI**
**Age**			
**Up to 35**	0.125	1.50	0.89–2.51
**36-69**	0.005	1.00	
**70 and above**	0.002	1.64	1.19–2.26
**Tumor size**			
**T1**	<0.001	1.00	
**T2**	0,027	1.60	1.05–2.43
**T3**	<0.001	2.31	1.41–3.80
**T4**	<0.001	4.34	2.77–6.79
**Lymph node status**			
**N0**	<0.001	1.00	
**N1**	0.005	1.75	1.18–2.60
**N2**	<0.001	2.73	1.82–4.09
**N3**	<0.001	3.59	2.35–5.48
**Histologic grade**			
**Grade 1**	<0.001	1.00	
**Grade 2**	0.313	1.27	0.80–2.01
**Grade 3**	<0.001	2.54	1.62–3.96

*HR* hazard ratio, *95% CI* 95%, Confidence interval.

When the basic cause of death, as classified by the state’s technician, was used alone, 25 patients (9.22% of the total of deaths) would have been censored and not considered to have died from breast cancer. In such cases, information contained in the death certificate suggested breast cancer as a contributing cause of death, and we decided to be conservative and, as done by other authors, consider the patient as having died from breast cancer [12]. These patients’ basic causes of death were: diseases of the circulatory system (7 cases, ICD chapter IX); endocrine, nutritional and metabolic diseases (3 cases, ICD chapter IV); diseases of the respiratory system (2 cases, ICD chapter X); diseases of the blood and blood-forming organs (1 case, ICD chapter III), and other neoplasms: unspecified malignant neoplasm of the liver (3 patients), unspecified malignant neoplasm of the bronchus and lung (3 cases), malignant neoplasm of the cerebellum (1 case, C71.6), malignant neoplasm of the cervix uteri (1 case, C53.9), malignant neoplasm of bone and articular cartilage of other and unspecified sites (1 case, C41.9), Letterer-Siwe disease (C96.0, 1 case), malignant neoplasm of the brain (1 case, C71.9), and malignant neoplasm of the mandible (1 case, C41.4). In those latter cases, the other cancer could have been the primary cause of death, but it seems more plausible, except for the patient who had a cervical cancer, that they were secondary malignancies.

When patients with deaths that were correctly classified as due to breast cancer were excluded (203 cases, 72% of total of deaths), higher stage (stage III versus stages I and II) remained associated with a higher HR of dying from other causes (HR = 2.02, CI 1.30-3.14, p = 0.002). After reading other death causes present in the death certificate and reassigning the basic death cause, this effect disappeared (p = 0.16).

## Discussion

Five-year breast cancer-specific survival for the entire cohort was 78.5%. Our survival findings are in accordance with earlier studies that were based on different Brazilian cohorts. The study by Ayala [13] described 5-year survival rates of 97% for stage I, 88% for stage II, and 51% for stage III in patients treated in the SUS, considering patients diagnosed at a similar period to the one of our study (2000–2009). Cintra *et al*. [14] showed a 5-year breast cancer-specific survival of 90% for stage I, 89% for stage II, and 68.7% for stage III patients from a mixed sample of the SUS and private systems treated from 1998 to 2000. Schneider & d’Orsi [12] showed survival proportions of 93.6% for stage I, 87.8% for stage II, and 62.5% for stage III patients, also from a mixed sample, diagnosed between 2000 and 2002. Menke *et al*. [24] showed an overall survival (all causes of death) above 80% in Porto Alegre, Rio Grande do Sul, in a study with patients treated from 1972 to 2002. In this study, the origin of the sample (SUS or private system) was not specified. Variations in survival could be due to different methodologies applied in each of the studies but also to different sample compositions regarding stage, age, and other biologic tumor factors, as well as differences in local cancer care.

For patients diagnosed in the United States in the years 2001 and 2002 (National Cancer Data Base), 5-year overall survival was 87.8% for stage I, ranged from 74% to 81.4% for stage II (IIB and IIA, respectively) and from 41% to 66.7% for stage III disease (IIIB and IIIA, respectively) [5]. In a public hospital in Barcelona, Spain, 5-year breast cancer-specific survival of patients diagnosed from 1992 to 2005 was 97.1% for stage I, 88% for stage II, and 70.1% for stage III patients [25].

Studying breast cancer survival and prognostic factors gives us insight into the natural history of the disease. Many prognostic factors have been studied over the years. The factor with the highest impact on survival is lymph node invasion (N). Tumor size (T) and distant metastasis (M) also play an important role, as well as lymph vascular invasion, positivity for hormone receptors, and over-expression of the HER2 protein [5]. Many other markers are linked to breast cancer survival [5]. In spite of the growing number of markers being discovered recently, the TNM remains the most important predictor of breast cancer survival [5]. In our study, tumor size and lymph node status were the strongest predictors of survival.

Socioeconomic status is also an intervening factor [6-8]. Most patients from our study were treated in the Brazilian public health system (SUS). Since lower income patients do not have private health insurance and usually cannot afford breast cancer treatment, they rely on the SUS for it. Not having private insurance and thus using the SUS was considered a surrogate for socio-economic information. The SUS provides multiple modalities of treatment for breast cancer patients, such as surgery and radio- and systemic therapy [10,11]. Our findings suggest that the survival of patients from the SUS is shorter than from the ones of the private system. Most of this difference is likely due to the different distribution of stages at diagnosis. Other contributing factors that were not analyzed in the present study could also explain this finding, such as larger interval between diagnosis and treatment in SUS’ patients [6,14], more difficult access to health care facilities, different comorbidities, smaller proportion of women undergoing screening, and a different lifestyle with other risk factors for death [8,9,26,27]. To minimize treatment delay, a federal law that was approved in 2012 stated that after diagnosis, cancer patients should be treated at an interval no longer than 60 days in the SUS [28].

The Brazilian SUS also provides breast cancer screening with mammography according to national guidelines [29]: since 2004, women aged 50–69 years have been encouraged to undergo mammography every 2 years, and also to have their breasts examined by a physician since 40 years of age. In private practice, guidelines from the Brazilian Society of Breast Surgery (Sociedade Brasileira de Mastologia) are followed, with a recommendation to use mammography screening yearly since 40 years of age [30]. In spite of these recommendations, Marchi and Gurgel [31] showed that women’s adherence to screening is low, with less than 50% performing biannual exams (24.5% for SUS patients and 42.9% for patients from the private system from 2003 to 2008). Another study showed similar results (34.9% adherence for women aged 50–59 years of the SUS and 71% for women of the private health system) [32]. Nevertheless, the use of mammograms is growing, with 54.6% of women 50 to 69 years of age having undergone at least one mammogram in their lifetime up to 2003 and 71.5% up to 2008 [33]. The proportion of women older than 70 years old undergoing mammography is smaller (37.1% up to 2003 and 54.5% up to 2008) [33]. Lower screening rates are consistently associated with not having private insurance and smaller income in many studies [31-34]. With the Brazilian Information System for Breast Cancer (Sistema de Informação do Câncer de mama - SISMAMA), implemented by the Brazilian National Cancer Institute in 2009, the number of women undergoing screening in the SUS is expected to rise. It will possibly result in more patients being diagnosed at earlier stages [29] and better overall survival. In our study, the frequencies of stages did not change over the years (P = 0.114, data not shown). It is possible that in the later years of the study, more patients were diagnosed with *in situ* tumors, which has been shown in a previous publication [18], but these tumors were not the scope of the present study. Also, our time span was too small to show any differences.

In our study, patients 70 years old and older had shorter breast cancer-specific survival. Schonberg *et al*. [35] showed a higher mortality for women older than 80 years in the US, and they argue that these women could have undergone less-than-standard treatment. This explanation has been presented by other authors and could have been the case for our patients [25,36]. Comorbidities can play a role, as well as smaller proportions of patients undergoing screening in this population [25,33]. Thus, our results differ from the findings of Brito *et al*. [6], which show better breast-cancer specific survival for patients older than 70 years treated in the SUS between 1999 and 2002 and shorter for younger patients (at the end of their study, 81.5% of patients older than 70 years were alive, versus only 45.4% of patients less than 35 and 72% for patients 35 or more and less than 70 years of age) [6]. On the other hand, older women are more likely to die of a variety of other causes, mainly cardiovascular diseases [26,37].

Patients up to 35 years of age were not more likely to die from breast cancer than patients 36–69 years of age. This could be due to our small number of cases at this age (only 47 women were younger than 35 years of age). These patients are unlikely to die from other causes when diagnosed with breast cancer [37,38]. Women with more advanced stages at diagnosis or recurrent disease are also more likely to die of breast cancer [23,37,38]. It is still debated whether younger age at diagnosis is an independent prognostic factor for shorter survival or if younger patients have tumors with worse biological features [37,39].

Our study has some limitations. First, the possibility of having wrongly classified a woman as being dead or alive exists, due to possible errors in the Mortality Information System. Three variables (patients’ names, mothers’ names and date of birth) were used in the record linkage to minimize this bias. Also, fathers’ names and patients’ addresses were used to confirm the pair as a true one. The medical records for a small sample of patients (70 cases, 0.08%) were checked. Only one patient was identified as having moved to another state, and since information on life status could be wrong, she was excluded from the study. Second, since high-quality data were only available in surgical treatment and neoadjuvant therapies, we chose not to include these variables in the multivariate Cox model, to avoid bias. The inclusion of patients who underwent neoadjuvant systemic therapies is unlikely to have affected our results; those patients had more advanced tumors at diagnosis and thus would very likely have undergone chemotherapy after surgery. Also, information on socioeconomic status, such as family income and educational level, were not available. Paim *et al.*[11] reported that having a private insurance is correlated with family income; thus, in our study, not having a private insurance was considered a surrogate for lower socioeconomic status.

On the other hand, our study also has strengths. Selecting patients from pathology reports has the advantage of providing good-quality data regarding stage, histologic tumor grade, and type. The information on histologic grade was missing in only 11 patients, either because the invasive component was too small (microinvasive tumor) or because the patient underwent neoadjuvant systemic therapy and the tumor sample prior to the systemic treatment was insufficient to assess histological grade. Even though we have limited information on treatment due to the origin of our data, this study brings insight into recent survival of women with operable breast cancer at a tertiary health facility that treats mostly low-income patients.

Different methods are used for survival analysis. Overall mortality, cause-specific mortality, and relative survival have all been used as endpoints [23,37,40]. The problem with the use of cause-specific mortality is the difficulty, in some cases, in attributing a death to breast cancer or its treatment [23,41]. For instance, some common sites for metastases of breast tumors can be reported as the primary site in death certificates, such as lung, bone, liver, and brain [41].

Cancer-specific survival depends on the data quality of death certificates, as well as in appropriate coding of reported causes of death. In Brazil, data quality has improved over the years [22], but still there are deaths of unknown causes or without medical assistance (2.8% of our cases). Moreover, even when death causes are cited in the death certificate, sometimes it is difficult to attribute a death to breast cancer or its treatment [17,23]. In our study, 25 deaths (9.22% of total of deaths) were not initially considered to be from breast cancer in the Mortality Information System. The cause reported by this system is the one considered in national mortality statistics; thus, wrongly assigning a cause could influence these indexes. On the contrary, all-cause mortality could result in underestimation of breast cancer survival [23,40]. Since we needed comparability with Brazilian cohorts, breast-cancer specific survival was used.

Different populations are subject to innumerous differences in life expectancy, life styles, and access to health care that could affect their survival, both from breast cancer and from other causes [4,6,9,23,27]. Trying to make comparisons among populations can help highlight these differences and guide local policies towards a more effective approach to breast cancer care, especially through earlier diagnosis and treatment of the disease [1,8,10,33]. For instance, in spite of not having addressed patients’ comorbidities, this study suggests that policymakers should pay attention to women older than 70 years; with screening, it is possible that they will be diagnosed with earlier tumors. Since age is the most important risk factor for breast cancer, and the Brazilian population is aging [11], this should be taken into account.

## Conclusions

In our study, 5-year breast cancer-specific survival was comparable to the one estimated for other Brazilian cohorts. Comparisons with estimates for high-income countries showed mixed results, which may be due to differences in the socioeconomic, demographic and health characteristics of the population subgroups analysed in each study. Also, patients treated by the SUS had a shorter survival rate than those treated through the private system, mostly due to higher initial stage of the disease. Patients older than 70 years had shorter survival time in comparison with patients 36–69 years of age. After reassigning the cause of death reported in the death certificate, more patients were considered to have died from breast cancer than when using only the basic cause of death, suggesting that one should be aware of the possible pitfalls of national cancer mortality statistics.

## Competing interests

The authors declare that they have no competing interests.

## Authors’ contributions

DB planned the study, gathered the sample, performed the record linkage, statistical analysis and wrote the manuscript. CMT planned the study, aided in the record linkage and statistical analysis and critically revised the manuscript. HG planned the study, analyzed all pathology samples and critically revised the manuscript. All authors approved the final version of the manuscript.

## Pre-publication history

The pre-publication history for this paper can be accessed here:

http://www.biomedcentral.com/1471-2407/13/434/prepub
